# Genomic and Transcriptomic Profiling of a Highly Virulent *Plesiomonas shigelloides* Strain: Insights into Pathogenicity and Host Immune Response

**DOI:** 10.3390/microorganisms13092168

**Published:** 2025-09-17

**Authors:** Zhixiu Wang, Shaoxuan Gu, Wen Lv, Jiayi Chen, Min Xue, Suli Liu, Jiaming Mao, Guohong Chen

**Affiliations:** College of Animal Science and Technology, Yangzhou University, Yangzhou 225009, China

**Keywords:** *Plesiomonas shigelloides*, genome sequencing, pathogenicity, histopathological analysis, transcriptome, immune responses

## Abstract

*Plesiomonas shigelloides*, a Gram-negative bacterium prevalent in aquatic environments and also frequently isolated from livestock and poultry, was investigated through integrated whole-genome sequencing and functional analyses. We deciphered the pathogenic mechanisms of *P. shigelloides* CA-HZ1, a highly virulent strain isolated from a novel piscine host, revealing a complete genome assembly with a 3.49 Mb circular chromosome and 311 kb plasmid housing 3247 predicted protein-encoding genes. Critical genomic features included 496 virulence factors and 225 antibiotic resistance genes. Pathogenicity analysis indicated that *P. shigelloides* was responsible for disease outbreaks. Antimicrobial susceptibility tests showed resistance to various drugs, such as kanamycin, erythromycin, and penicillin. Histopathological examination showed significant alterations in the infected hosts. Quantitative real-time PCR (qRT-PCR) was carried out to analyze immune-related gene (IL-6, IL-1β, IL-21, STAT1, and HSP70) levels in liver and intestinal tissues, demonstrating the potent immunity triggered by *P. shigelloides* infection. An analysis of the liver transcriptome revealed that *P. shigelloides* has the potential to influence the cellular composition, molecular functions, and biological processes. Collectively, this study describes the genomic basis underlying both the pathogenic potential and hypervirulence of *P. shigelloides* CA-HZ1, establishing a foundational framework for investigating its broad host tropism and immune response.

## 1. Introduction

*Plesiomonas shigelloides* is a motile, rod-shaped, non-spore-forming, Gram-negative, facultatively anaerobic bacterium within the family Enterobacteriaceae [1,2]. It predominantly colonizes freshwater and estuarine ecosystems [3]. *P. shigelloides* has been credited with causing many infections over the years [4]. Additionally, it demonstrates a broad host tropism, exhibiting pathogenic potential in aquatic species and poultry besides mammals, especially in geese [5]. Critically, not all *P. shigelloides* strains are pathogenic; as an opportunistic pathogen, its virulence manifestation requires a confluence of three synergistic factors: compromised host immunity, permissive environmental conditions, and strain-specific virulence determinants. *P. shigelloides* has been isolated from multiple aquaculture species as reported in aquatic animals. Studies of intestinal microbiota in freshwater teleosts reveal that *Plesiomonas*, alongside *Fusobacteria* and *Aeromonas*, constitutes one of the predominant genera within the bacterial communities of these vertebrates [6]. Notably, recent studies have demonstrated pathogenic *P. shigelloides* infections in multiple cultured fish species, including grass carp (*Ctenopharyngodon idella*) [7], common carp (*Cyprinus carpio*) [8], silver carp (*Hypophthalmichthys molitrix*) [9], and Chinese sturgeon (*Acipenser sinensis*) [10]. The northern snakehead (*Channa argus*) is a predatory freshwater fish from the family Channidae, which is native to tropical Asia and Africa [11]. In China, it holds significant commercial value due to its rapid growth, high-quality meat, and anti-inflammatory and therapeutic properties [12]. As the scale and density of farming increase, diseases in *C. argus* have become more prevalent, posing a threat to the aquaculture industry. Pathogens affecting *C. argus* include fungi, bacteria, parasites, and viruses [13]. However, there is currently a lack of research on diseases caused by *P. shigelloides* in *C. argus*, and their interactions remain largely unexplored.

The pathogenicity of bacterial organisms is governed by a complex arsenal of molecular determinants that enable them to colonize, invade, evade host immunity, and cause damage. A variety of virulence factors have been associated with *P. shigelloides* infection, including hemolysin, enterotoxins, cholera-like toxins, lipopolysaccharide (LPS), and iron acquisition systems, among others. However, genomic analyses indicate that this bacterium exhibits a high rate of genetic recombination, which may contribute to genetic diversity and variations in virulence [14]. Studies by Yaikhan et al. have shown that virulence factors associated with flagellar assembly and motility enhance the ability of *P. shigelloides* to colonize the human intestinal tract and cause disease [5]. However, significant variations have been observed in the activities of elastase, protease, histidine decarboxylase, and hemolysin among different strains [15]. *P. shigelloides* strains isolated from different countries and sources, such as humans, animals, and the environment, exhibit high biochemical and serotypic diversity. Pathogenicity-related traits, including motility, DNase activity, gelatinase activity, and hemolytic activity, are unevenly distributed among strains from various origins [16]. Despite the clinical significance of *P. shigelloides*, a comprehensive and mechanistic understanding of its virulence arsenal remains fragmented. As a bacterium widely distributed in aquatic ecosystems, its pathogenic potential is complex and difficult to predict. *P. shigelloides* possesses multiple virulence factors that may contribute to its mechanisms of pathogenicity, but its ability to cause disease likely depends on the combined effects of various factors and host status, highlighting its complex potential as a waterborne and foodborne pathogen.

This study establishes the inaugural documentation of *P. shigelloides* pathology in a novel piscine model, successfully isolating and characterizing the hypervirulent strain CA-HZ1. The study performed whole-genome sequencing and analysis of the strain, assessed its antimicrobial susceptibility and pathogenic potential, and examined histopathological alterations in infected hosts. This approach was integrated with transcriptomic profiling to decipher host–pathogen interactions. Collectively, investigation of this strain provides insights into its ecological role, adaptability to environmental changes, and potential contribution to biodiversity. These findings unravel fundamental mechanisms of *P. shigelloides* virulence and immune modulation, providing a foundation for developing broad-spectrum interventions against *P. shigelloides* infections.

## 2. Materials and Methods

### 2.1. Bacterial Isolation and Identification

This study isolated the *P. shigelloides* strain in farmed *C. argus* in Huzhou City, Zhejiang Province, China. Moribund *C. argus* underwent sanitization using 75% ethanol before dissection. Liver, kidney, and spleen tissues were inoculated on Luria broth (LB) nutrient agar plates using a sterile loop. Following 16 h incubation under 28 °C, we purified the predominant uniform isolates. The supernatant was eliminated after 3–5 min of bacterial solution centrifugation at 8000 rpm; thereafter, the pellet was dehydrated with ethanol and fixed with 2.5% glutaraldehyde for scanning electron microscopy. Gram staining was performed by standard protocols, and growth curve analysis was conducted in LB broth at 28 °C with continuous shaking (150 rpm). The purified strain was preserved in 20% (*v*/*v*) glycerol at −40 °C.

MolPure^®^ Bacterial DNA Kit (Yeasen Biotechnology, Shanghai, China) was employed for extracting DNA in the *P. shigelloides* strain. After inoculation in the LB liquid medium with 1% content, bacterial incubation at 150 rpm and 28 °C on a shaker was completed. Optical densities at 600 nm (OD_600_) were measured hourly for 24 h. 16S rRNA and the gyrB gene were amplified through PCR, and sequence homology was analyzed using the BLAST algorithm (Basic Local Alignment Search Tool) on the NCBI platform (https://blast.ncbi.nlm.nih.gov/Blast.cgi, accessed on 15 May 2025). Additionally, the neighbor-joining method was adopted for constructing phylogenetic trees with the MEGA software package.

### 2.2. Physicochemical Property Measurement and Antibiotic Susceptibility Test

After isolation, we inoculated bacteria in biochemical tubes to characterize their physicochemical properties. Specific biochemical assays were performed following the standard procedures outlined in the instructions (Hangzhou Microbial Reagents Co., Ltd., Hangzhou, China). ATCC 14029 (ATCC, Manassas, VA, USA) was used as a reference strain. Combined with the physicochemical profiling, molecular identification based on 16S rRNA/gyrB gene sequencing and phylogenetic analysis provided definitive species assignment, which was subsequently cross-verified using Bergey’s Manual of Systematic Bacteriology. The phylogenetic tree was constructed using the neighbor-joining (NJ) method in MEGA 5.0 software. The Kirby–Bauer test was used to determine the bacterial susceptibility to 35 antimicrobial drugs (Hangzhou Microbial Reagents Co., Ltd., Hangzhou, China) (CLSI-M100).

### 2.3. Whole-Genome Sequencing and Genome Annotation

Complete bacterial genomes were sequenced using a hybrid approach combining PacBio and Illumina sequencing technologies. Filtered clean reads meeting quality thresholds were subjected to de novo assembly to generate genome sequences. Final assemblies were produced with Unicycler v0.4.8 for third-generation sequencing, with sequence correction implemented via Pilon v1.22 during the assembly process. Plasmid identification was performed using PlasFlow (https://github.com/smaegol/PlasFlow, accessed on 15 May 2025) on genome assemblies. Resulting plasmid sequences were annotated through BLAST searches against the PLSDB database (https://ccb-microbe.cs.uni-saarland.de/plsdb/, accessed on 15 May 2025). Coding sequences (CDSs) were predicted using Glimmer (http://ccb.jhu.edu/software/glimmer, accessed on 15 May 2025), GeneMarkS, and Prodigal. Functional annotations were generated from five databases: NR, SwissProt, Pfam, eggNOG, and Gene Ontology (GO). Genome circle plots were visualized using Circos on cloud computing platforms. Virulence and resistance gene prediction was performed using the Virulence Factor Database (VFDB; http://www.mgc.ac.cn/VFs, accessed on 15 May 2025) and the Comprehensive Antibiotic Resistance Database (CARD; http://arpcard.mcmaster.ca, v1.1.3, accessed on 15 May2025).

### 2.4. Bacterial and Fish Preparation

This study inoculated the *P. shigelloides* strain in LB broth medium. After 16 h of culture within the 28 °C shaker incubator, the broth culture experienced 15 min of centrifugation at 8000× *g* and 4 °C for precipitate collection. The bacterial concentration (colony-forming units per milliliter, CFU/mL) was determined by standard plate counting. Serial ten-fold dilutions of the washed bacterial suspension were prepared in sterile PBS. Aliquots (100 µL) of appropriate dilutions were spread onto LB agar plates. Colonies were counted, and the CFU/mL of the stock suspension was calculated based on plates yielding 30–300 colonies. After rinsing thrice using sterile phosphate-buffered saline (PBS, pH 7.4, 0.01 M), the bacterial pellet underwent resuspension within PBS at 2.16 × 10^8^ CFU/mL. We obtained normal *C. argus* whose mean body weight was 15.39 ± 2.31 g from a fish farm in Huzhou, Zhejiang. Prior to experimentation, randomly selected fish underwent comprehensive health screening, including visual inspection for dermal lesions, ectoparasites, and gill abnormalities, and bacteriological screening via culture isolation to exclude bacterial pathogens (e.g., *Aeromonas* spp. and *Streptococcus* spp.). Molecular confirmation of viral pathogen absence was carried out through PCR amplification of target genes (e.g., LMBV), with concurrent monitoring of water quality parameters: dissolved oxygen > 6 mg/L, pH 7.2–7.6, and ammonia < 0.05 mg/L. During the 2-week acclimation period at 25 ± 0.5 °C, fish were fed twice daily with a commercial diet (HongLi, Hangzhou, China) at 3.0% of total body weight per feeding.

### 2.5. Infection and Sample Collection

Before the challenge, 20 fish were selected again at random and confirmed as free from *P. shigelloides* or additional pathogens through isolating and identifying bacteria. To statistically analyze mortality, we randomly classified fish into six groups of 50 *C. argus* each and injected them intraperitoneally using 100 µL *P. shigelloides* at varying concentrations ranging from 2.16 × 10^4^ CFU/mL to 2.16 × 10^8^ CFU/mL. PBS was injected into the control group. Mortality was recorded daily over seven days. The median lethal dose (LD_50_) was calculated using the modified Reed–Muench method, and the 7-day median lethal concentration (LC_50_) was computed via trimmed Spearman–Karber analysis. In addition, to analyze immune response, we randomly classified fish as 2 groups (*n* = 150 each). Meanwhile, 0.1 LD_50_ (100 µL) was injected into the infection group, whereas PBS was given to the control group. Liver samples were collected at 1, 2, and 3 days post-infection (dpi). The samples were collected from five fish, with 3 replicates per time point. We balanced the sex ratio in each replicate through random selection to minimize any potential bias related to sex/age. After liquid nitrogen freezing, tissues were preserved under −80 °C. Prior to all piscine surgical procedures, including bacterial isolation, tissue sampling, and injection-based infections, tricaine methanesulfonate (MS-222) was administered via immersion anesthesia to ensure compliance with animal welfare protocols. All experimental protocols gained approval from the Animal Experiment Ethics Committee of Yangzhou University (permit number: SYXK(Su) IACUC 2018-0017, approval date: 6 January 2018).

### 2.6. Histopathological Analysis

Prior to sampling, tricaine methanesulfonate (MS-222) anesthesia was administered to ensure compliance with animal welfare protocols. The liver, kidney, spleen, and intestine were removed and preserved with 4% formaldehyde for histological examinations. Then, they were dehydrated with gradient ethanol to absolute ethanol, followed by paraffin embedding and sectioning using traditional histological methods. The sections underwent hematoxylin and eosin (H&E) staining, followed by examination with a light microscope [17].

### 2.7. Reverse Transcription Quantitative PCR (RT-qPCR) and Statistical Analysis

Total RNA was extracted from tissues using RNAiso Plus (Takara Bio, Dalian, China, Cat# 9109) according to the manufacturer’s instructions, followed by DNase I treatment (Cat# EN0521,Thermo Fisher Scientific, Waltham, MA, USA) at 37 °C for 30 min. cDNA was synthesized from 1 µg DNase-treated RNA using the TransScript One-Step gDNA Removal and cDNA Synthesis SuperMix (Cat# AT311-03,TransGen Biotech, Beijing, China) according to the protocol. cDNA concentration was quantified via a Qubit 4.0 Fluorometer (Thermo Fisher) and diluted to 25 ng/µL in nuclease-free water (Ambion, Austin, TX, USA) for standardization. Five immune-related genes (*IL-6*, *IL-1β*, *IL-21*, *STAT1*, and *HSP70*) were selected to evaluate the immunity of *C. argus* against *P. shigelloides* infection. Table 1 displays primers utilized in amplification. The specific primers were successfully developed based on conserved regions of target gene sequences retrieved from GenBank using Primer Premier 5.0. All assays were carried out thrice, with β-actin gene expression being the endogenous reference. qPCR was conducted within the 20 μL volume: SYBR^®^ SuperMix (Bio-Rad Laboratories, Hercules, CA, USA), 10 μL; forward and reverse primers (0.4 μM), 1 μL each; diluted cDNA, 1 μL; and nuclease-free water, 7 μL. The PCR program included 10 min initial denaturation at 95 °C, 10 s at 95 °C, and 30 s at 60 °C for 40 cycles. Biological replicates (*n* = 5 fish/group) with triplicate technical runs per sample were conducted. The β-actin served as an endogenous reference, with stability validated using NormFinder (ΔCq SD < 0.5 across groups). The 2^−ΔΔCT^ approach was employed for calculating relative gene levels. Results were indicated by means ± standard error (SE) of 3 separate assays. One-way ANOVA was used for statistical analysis (*p* > 0.05, non-significance; *p* < 0.05 (*), significance; and *p* < 0.01 (**), extreme significance).

### 2.8. Transcriptome Sequencing and Analysis

To perform transcriptome sequencing, we also randomly classified fish as 2 groups (*n* = 60 each). Meanwhile, 0.1 LD_50_ (100 µL) was injected into the infection group, whereas PBS was given to the control group. Liver samples were collected at 1 and 3 dpi. The samples included tissues collected from five fish, with 3 parallel samples at each time point. After liquid nitrogen freezing, tissues were preserved under −80 °C. For sample processing and animal welfare, refer to Section 2.5. After extracting total RNA from the samples, a NanoDrop 2000 spectrophotometer was utilized to assess the purity and integrity. Using magnetic beads with Oligo (dT), mRNA was isolated. It was then broken up into little pieces and transformed into single-stranded cDNA using random primers. A SuperScript double-stranded cDNA synthesis kit was used to create double-stranded cDNA. The generated cDNA underwent end repair, phosphorylation, and adaptor insertion following the library creation technique. Libraries were size-selected for 300 bp cDNA target fragments on 2% LowRange Ultra Agarose. Phusion DNA polymerase (NEB) was then used for 15 cycles of PCR amplification. Qubit 4.0 was used for quantification of the sequencing library. The NovaSeq Reagent Kit was used for sequencing on the NovaSeq X Plus platform (PE150). Differentially expressed genes (DEGs) were analyzed using DESeq2 to compare two groups, with a false discovery rate of <0.05 and a threshold for significantly DEGs set at |log2 (fold change)| of ≥1. The highly enriched DEGs in Gene Ontology (GO) (http://geneontology.org/, accessed on 1 June 2025) terms and metabolic pathways were then found using functional classification and enrichment analysis, which included GO and Kyoto Encyclopedia of Genes and Genomes (KEGG) (https://www.genome.jp/kegg/, accessed on 1 June 2025). A Bonferroni-corrected *p*-value of less than 0.05 was used for this study in comparison to the whole-transcriptome background. The Goatools (https://github.com/tanghaibao/goatools, accessed on 1 June 2025) and Python scipy programs (https://scipy.org/, accessed on 1 June 2025) were used for KEGG pathway analysis and GO functional enrichment, respectively.

## 3. Results

### 3.1. Bacterial Isolation and Identification

**Figure 1:**
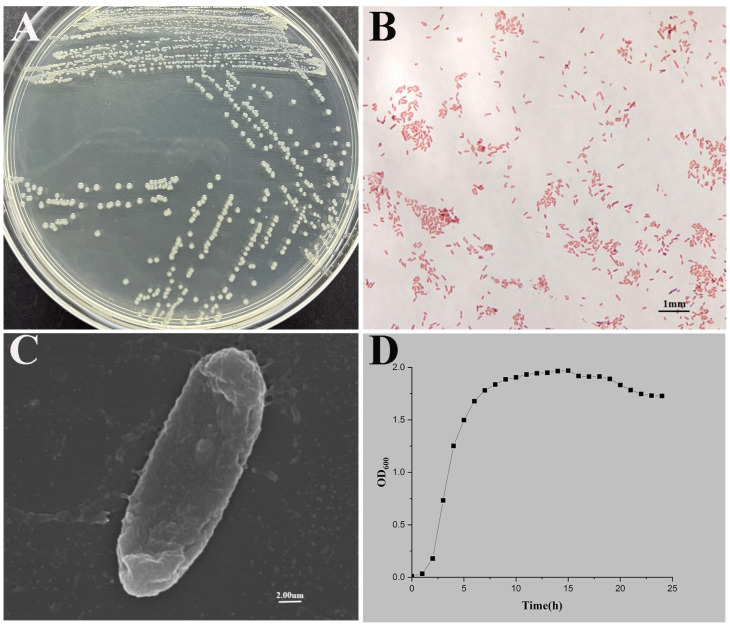
*P. shigelloides* strain CA-HZ1 isolation and identification. (**A**) Colony morphology on LB agar. (**B**) Gram staining for strain CA-HZ1 under light microscopy. (**C**) Scanning electron microscopy (20,000×). (**D**) Growth curves of strain CA-HZ1 at 28 °C.

**Figure 2:**
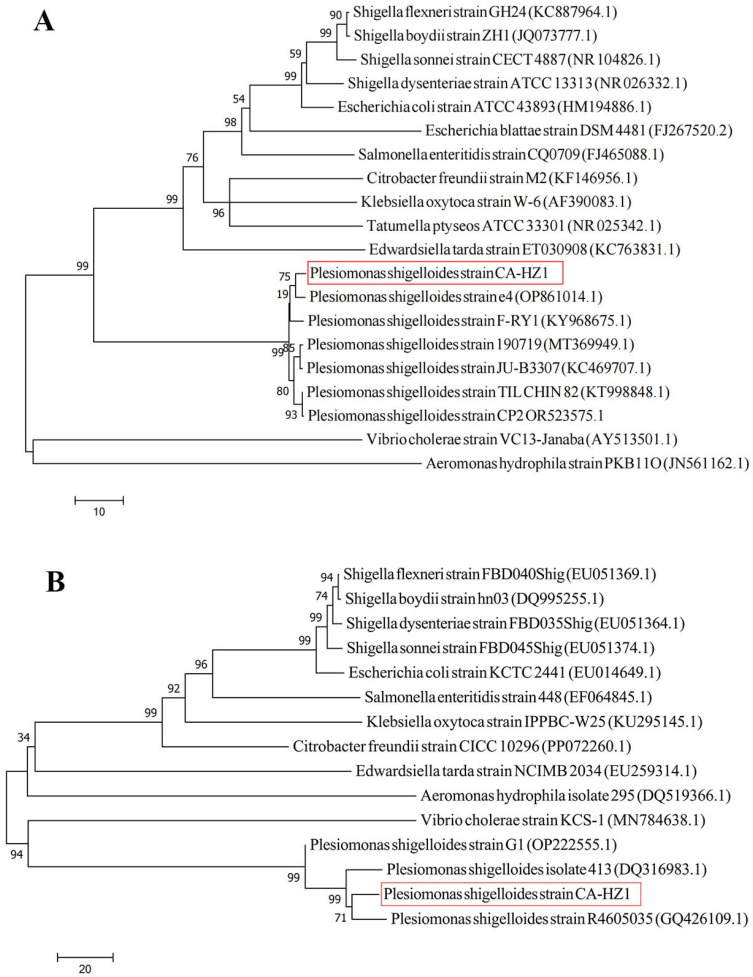
(**A**) Phylogenetic trees according to 16S rRNA sequence. (**B**) Phylogenetic trees according to gyrB sequence. Numerals at nodes are indicative of bootstrap percentages obtained based on 1000 replications. Numbers after species names represent GenBank accession numbers. The *P. shigelloides* CA-HZ1 strain is highlighted with a red frame in the figure.

Colonies on LB plates were circular, convex, and whitish, with Gram staining indicating a Gram-negative rod (Figure 1A,B). Scanning electron microscopy revealed representative rod-shaped bacteria that had complete margins (Figure 1C). The growth curve of *P. shigelloides* exhibited a lag phase during the initial 2 h post-inoculation, followed by exponential growth from 2 to 7 h (Figure 1D). As revealed by phylogenetic trees from 16S rRNA sequence analysis, the isolated bacteria clustered with known *P. shigelloides* strains (Figure 2A). Similarly, the phylogenetic tree constructed using gyrB sequences confirmed the bacteria as *P. shigelloides* (Figure 2B). The isolated *P. shigelloides* strain was designated CA-HZ1.

### 3.2. Physicochemical Characteristics of the Isolated Bacteria

Physiological and biochemical analyses revealed that the CA-HZ1 isolate tested negative for mannitol, arabinose, hydrogen sulfide, esculin, citrate, malonate, the V-P test, L-rhamnose, sucrose, D-xylose, dulcitol, arabinitol, sorbitol, and urea. The strain was positive for glucose, maltose, inositol, nitrate reduction, oxidase, and indole, preliminarily identifying it as *P. shigelloides* (Table 2).

### 3.3. Antibiotic Susceptibility Test

The drug-sensitive disk approach was adopted for assessing the isolated strain CA-HZ1’s susceptibility to 35 antibacterial drugs. Table 3 displays the results. This bacterium showed sensitivity to 24 antibiotics, including gentamicin, tobramycin, and norfloxacin, and resistance to 8 antibiotics, including kanamycin, erythromycin, and medemycin, as well as moderate sensitivity to amikacin, streptomycin, and ceftriaxone (Table 3).

### 3.4. Whole-Genome Sequencing and Genome Annotation

**Figure 3:**
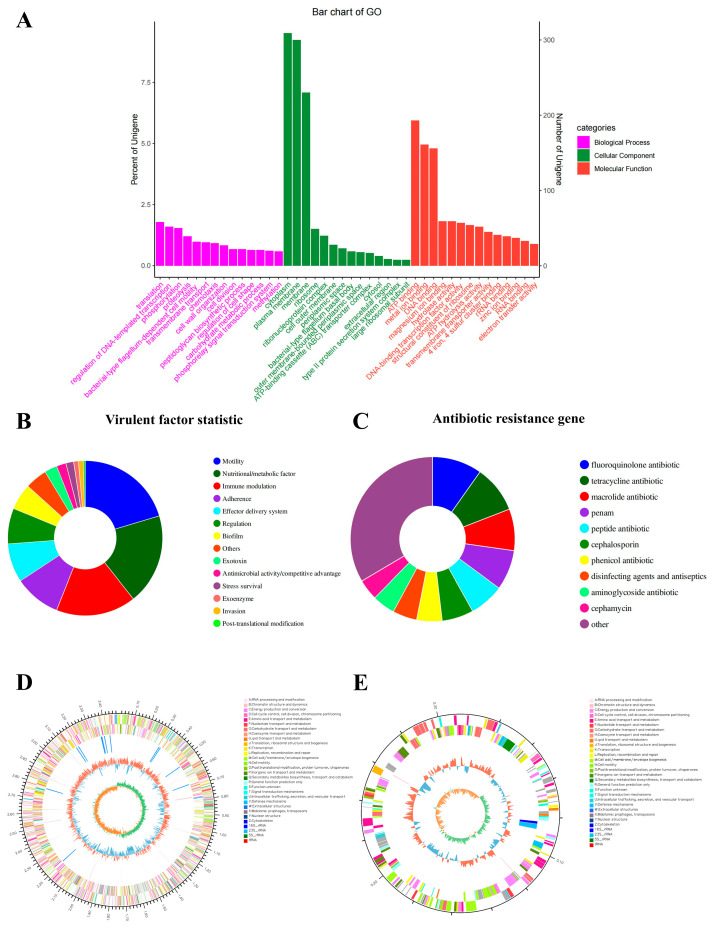
Whole-genome sequencing and genome annotation. (**A**) Gene Ontology (GO) slim classification. (**B**) Virulent factor. (**C**) Antibiotic resistance gene. (**D**) Chromosome Circos map of *P. shigelloides* CA-HZ1. (**E**) Circos diagram of plasmid architecture.

The whole genome of *P. shigelloides* strain CA-HZ1 was sequenced and annotated. In order to assess the biological relevance of the *P. shigelloides* CA-HZ1 gene pool, the genes were categorized by GO analysis based on matches to sequences of known function into three categories: biological process, cellular component, and molecular function (Figure 3A). There was a total of 496 virulent factors and 225 antibiotic resistance genes (Figure 3B,C; Tables S1–S3). The complete genome of *P. shigelloides* CA-HZ1 contained a circular 3,493,313 bp chromosome and a 311,332 bp plasmid (Figure 3D,E). Its chromosome contained 3247 predicted protein-encoding genes.

### 3.5. Pathogenicity Analysis

**Figure 4:**
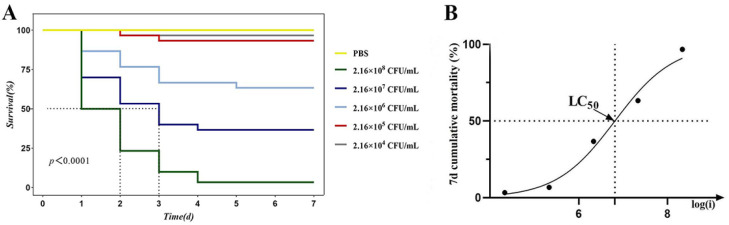
(**A**) Survival curves of *C. argus* after *P. shigelloides* infection at diverse concentrations. PBS at an equivalent amount was injected into the control group. (**B**) The 7-day LC50 curve based on survival results at different concentrations (i: diverse *P. shigelloides* strain gradient concentrations of 2.16 × 10^4^ to 2.16 × 10^8^ CFU/mL).

We analyzed cumulative survival rates in control and infection groups (Figure 4A). The mortality of the infection group was observed at 1 dpi under the greatest concentration gradient, with mortality increasing from 50% to 96.7% during 1–4 dpi. At the injection titer exceeding 2.7 × 10^7^ CFU/mL, mortality surpassed 50% in every group. The 7-day lethality profile of *P. shigelloides* in *C. argus* yielded LD_50_ (Figure 4A) and LC_50_ (Figure 4B) values of 1.83 × 10^4^ CFU/g and 2.22 × 10^6^ CFU/mL, respectively.

### 3.6. Histopathological Analysis

**Figure 5:**
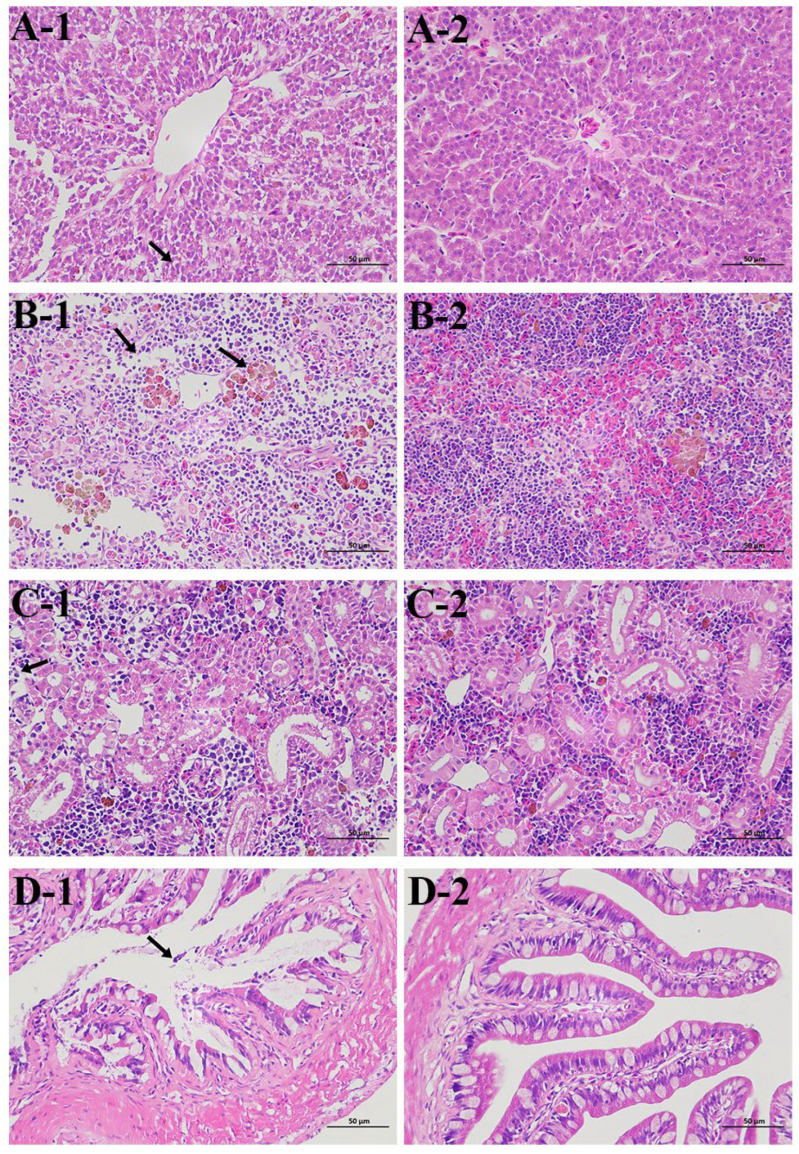
Histological sections for tissues from *C. argus* after *P. shigelloides* infection. (**A-1**) Infected liver (black arrow indicates loose cytoplasm in hepatocytes). (**A-2**) Uninfected liver. (**B-1**) Infected spleen (black arrow indicates decreased lymphocytes). (**B-2**) Uninfected spleen. (**C-1**) Infected kidney (black arrow indicates decreased lymphocytes). (**C-2**) Uninfected kidney. (**D-1**) Infected intestine (black arrow indicates shedding of epithelial cells in the mucosal layer). (**D-2**) Uninfected intestine; scale bar = 100 μm.

There were significant histopathological alterations seen among *P. shigelloides*-challenged moribund fish (Figure 5). *C. argus* under experimental infection were examined, which showed hepatocytes with loose cytoplasm, reduced lymphocytes in spleen and kidney tissues, and exfoliation of epithelial cells.

### 3.7. Liver Immune-Related Gene Expression Levels

**Figure 6:**
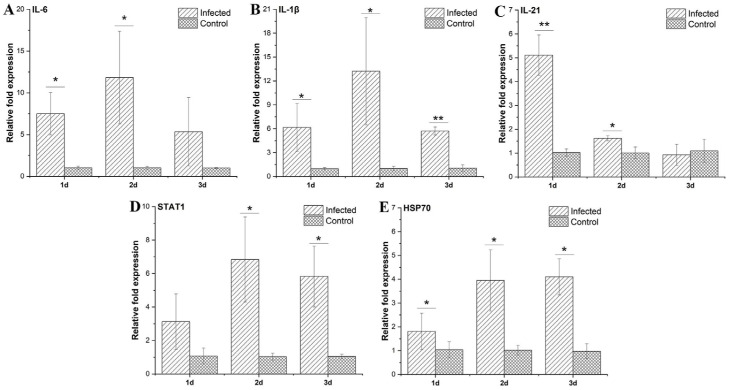
Immune-related gene patterns in *C. argus* liver at diverse times following *P. shigelloides* infection detected through qRT-PCR. (**A**) *IL-6*, (**B**) *IL-1β*, (**C**) *IL-21*, (**D**) *STAT1*, (**E**) *HSP70*. One-way ANOVA was used for statistical analysis (*p* > 0.05, non-significance; *p* < 0.05 (*), significance; *p* < 0.01 (**), extreme significance).

Immune gene expression patterns within tissues were measured through qRT-PCR to reflect the degree of immune response post-infection. There were obvious alterations of immune-related genes within liver tissues collected from *P. shigelloides*-infected *C. argus* in comparison with control fish (Figure 6). *IL-21* expression levels peaked at 1 dpi (Figure 6C). *IL-6*, *IL-1β*, and *STAT1* expression levels rapidly increased post-infection, peaking at 2 dpi with 11.84-fold, 13.22-fold, and 6.84-fold (all *p* < 0.05) elevation, respectively. Although levels decreased at 3 dpi, they remained higher than control levels (Figure 6A,B,D). *HSP70* expression continued to increase post-infection, peaking at 3 dpi with a 4.10-fold (*p* < 0.05) increase (Figure 6E).

### 3.8. Transcriptome Sequencing and Analysis of DEGs

**Figure 7:**
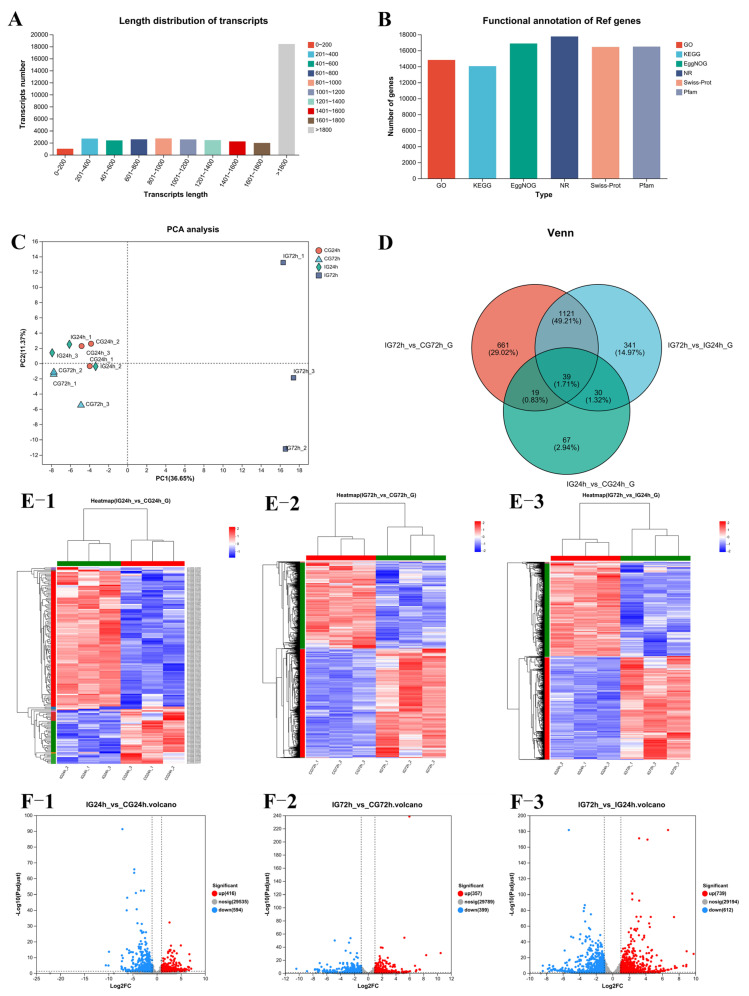
(**A**) Sequence length distribution. The length range of unigenes is represented by the abscissa; the number of unigenes within this range is shown by the ordinate. (**B**) Statistics on functional annotations for six distinct databases. The database name is shown by the abscissa, and the number of sequences annotated to the database is shown by the ordinate. (**C**) PCA of gene expression in different groups. (**D**) Venn diagram of the DEGs. (**E**) Cluster analysis of gene expression patterns: (**E-1**) IG24h vs. CG24h; (**E-2**) IG72h vs. CG72h; (**E-3**) IG72h vs. IG24h; (**F**) Volcano plot of differentially expressed genes; (**F-1**) IG24h vs. CG24h; (**F-2**) IG72h vs. CG72h; (**F-3**) IG72h vs. IG24h.

Over 5.89 GB of clean data were produced by each sample, yielding a total of 77.23 GB of clean data. The Q30 value exceeded 96.31% in all groups. Sequences longer than 1800 bp were the most abundant, while those between 0 and 200 bp were the least prevalent (Figure 7A). Six functional databases were used to classify these unigenes functionally, resulting in the annotation of 14,812 GO (83.41%), 14,038 KEGG (79.06%), 16,875 COG (95.03%), 17,754 NR (99.98%), 16,437 Swiss-Prot (92.57%), and 16,481 Pfam (92.81%) unigenes (Figure 7B). Pairwise comparisons between groups were conducted to identify DEGs. To illustrate the shared and unique DEGs among various groups, a Venn diagram was created (Figure 7C). The IG72h vs. CG72h, IG72h vs. IG24h, and IG24h vs. CG24h groups were revealed to have 661, 341, and 67 unique DEGs, respectively. Principal component analysis (PCA) was used to compare the transcriptomes of the four groups (Figure 7D). The findings demonstrated that 72 h after infection, the infected and control groups were well separated. Gene expression levels in the samples are shown in the cluster analysis diagram, where red indicates upregulated genes and blue indicates downregulated genes (Figure 7E). The results revealed 155 DEGs in the IG24h vs. CG24h comparison, comprising 110 upregulated genes and 45 downregulated genes (Figure 7(F-1)). A total of 1840 DEGs were found in the IG72h vs. CG72h comparison, comprising 1021 upregulated and 819 downregulated genes (Figure 7(F-2)). Additionally, a comparison of IG72h vs. CG72h revealed 1531 DEGs, of which 799 were upregulated and 732 were downregulated genes (Figure 7(F-3)).

### 3.9. GO Enrichment Analysis of DEGs

**Figure 8:**
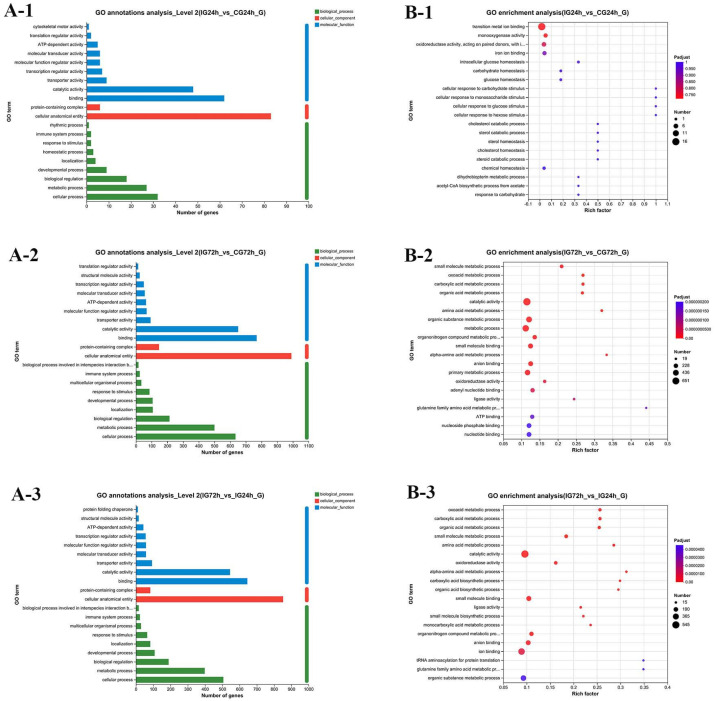
GO term. (**A**) GO classification of DEGs: (**A-1**) IG24h vs. CG24h; (**A-2**) IG72h vs. CG72h; (**A-3**) IG72h vs. IG24h. (**B**) Bubble diagram of the TOP 20 significantly enriched GO terms: (**B-1**) IG24h vs. CG24h; (**B-2**) IG72h vs. CG72h; (**B-3**) IG72h vs. IG24h.

The GO annotations analysis revealed involvement in three main sections: biological process (BP), cellular component (CC), and molecular function (MF). Among these, the subcategories with the highest enrichment rates were cellular process, binding, and cellular anatomical entity (Figure 8A). Figure 8B displays the top 20 GO terms that have been enhanced with DEGs. In the comparison between the IG24h vs. CG24h groups, the top 20 enriched terms comprised 4 MF and 16 BP categories, with the transition metal ion binding term having the largest number of DEGs (Figure 8(B-1)). The GO enrichment analysis results for the IG72h vs. CG72h group indicated that the DEGs were predominantly enriched in metabolic process, catalytic activity, primary metabolic process, and organic substance metabolic process (Figure 8(B-2)). The DEGs had high enrichment in ion binding, catalytic activity, and the organic substance metabolic process in the comparison between IG72h and IG24h, with all enrichment results being statistically significant (Figure 8(B-3)).

### 3.10. KEGG Pathways Enrichment Analysis of DEGs

**Figure 9:**
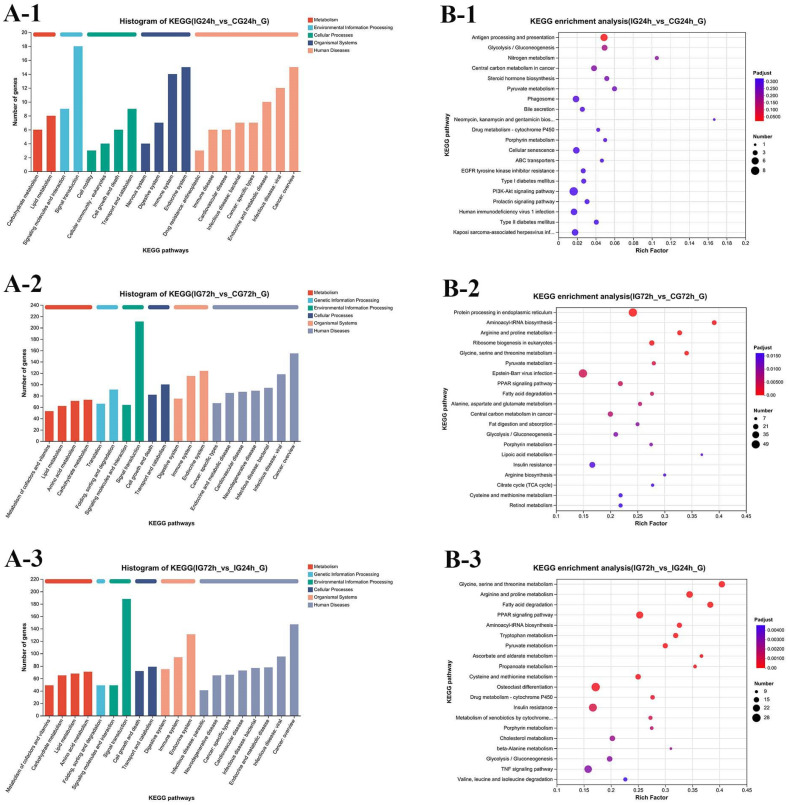
KEGG pathway. (**A**) KEGG pathway enrichment analysis of the DEGs: (**A-1**) IG24h vs. CG24h; (**A-2**) IG72h vs. CG72h; (**A-3**) IG72h vs. IG24h. (**B**) Bubble diagram of the TOP 20 significantly enriched KEGG pathways: (**B-1**) IG24h vs. CG24h; (**B-2**) IG72h vs. CG72h; (**B-3**) IG72h vs. IG24h.

Five categories were used to classify the KEGG annotations for comparison between the IG24h vs. CG24h groups: organismal systems, environmental information processing, cellular processes, metabolism, and human diseases. The subcategories with the highest enrichment rates were lipid metabolism, signal transduction, and cancer, which were the subcategories showing the greatest rates of enrichment (Figure 9(A-1)). The PI3K-Akt signaling pathway had the most DEGs according to the enrichment analysis, while the antigen processing and presentation pathway showed a significant difference (Figure 9(B-1)). For comparison between the IG72h vs. CG72h groups, KEGG annotations were categorized into six categories, with an additional category of genetic information processing compared to the IG24h vs. CG24h group. Human disease pathways had the highest number of annotated genes (Figure 9(A-2)). The enrichment analysis results indicated that the protein processing in the endoplasmic reticulum pathway had the largest number of DEGs and the most significant enrichment level (Figure 9(B-2)). Furthermore, the KEGG annotations for the IG72h vs. IG24h group were classified into six categories, with all 20 pathways showing significant enrichment (Figure 9(A-3,B-3)).

### 3.11. Analysis of KEGG Pathway for DEGs Associated with Immunity

**Figure 10:**
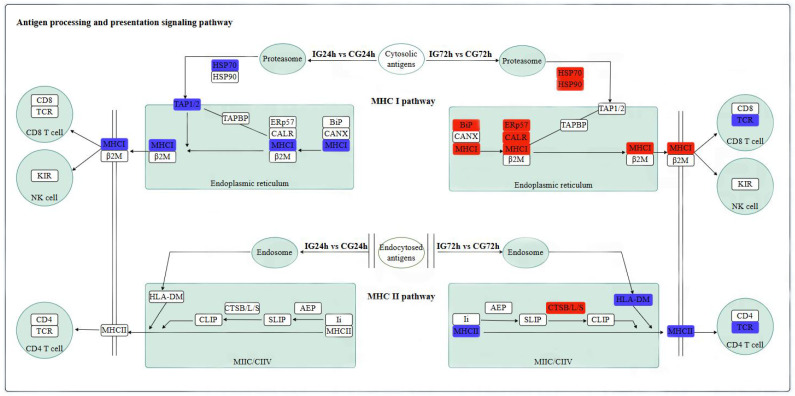
Antigen processing and presentation signaling pathway. The red boxes represent upregulated genes, and the green boxes represent downregulated genes.

In *P. shigelloides*-infected *C. argus*, DEGs were predominantly enriched in innate immunity pathways, particularly genes involved in PRR-mediated signaling cascades, including MAPK, antigen processing and presentation, and PI3K-Akt pathways. KEGG enrichment analysis confirmed significant activation of antigen processing and presentation pathway-associated genes at both 24 h and 72 h post-infection (Figure 10). This pathway can be divided into two subpathways: the MHCI pathway and the MHCII pathway. There were six DEGs related to antigen processing and presentation at 24 h after infection, including HSP70, TAP1/2, MHCI, etc. Among them, TAP plays a central role in MHC I antigen presentation, while HSP70 acts in an ATP-dependent manner and has been shown to be involved in immune stimulation, stress tolerance, and defense against bacterial invasion. There were 20 DEGs related to antigen processing and presentation at 72 h after infection, including MHCII, HSP90, CALR, BiP, CTSB/L/S, etc. Both the REP57 and CALR genes are present in the endoplasmic reticulum. ERP57 is involved in the assembly of major histocompatibility complex (MHC) class I molecules and regulates the immune response. These DEGs indicate that *P. shigelloides* invasion triggers activation of the antigen processing and presentation pathway, a core element of the robust innate immune response mounted by *C. argus*.

## 4. Discussion

*P. shigelloides* is a facultatively anaerobic, motile, Gram-negative bacillus. This bacterium occupies diverse ecological niches, including freshwater systems, surface water bodies, and wild and farmed animals. Its capacity to cause a spectrum of enteric infections has positioned it as a significant focus in global microbiological research. As a foodborne pathogen, *P. shigelloides* causes bacterial gastroenteritis in humans, with particularly high incidence in Southeast Asia and Africa, where infections are strongly associated with consuming raw seafood and contaminated water. Clinical manifestations range from acute secretory diarrhea to dysentery-like hemorrhagic diarrhea and chronic diarrhea; rarely, it may lead to sepsis, central nervous system infections, and ocular infections. While its pathogenic mechanisms remain incompletely understood, an outbreak of collective gastroenteritis in Anji County (Huzhou City, China, 2023) traced to *P. shigelloides* contamination of freshwater environments demonstrates its zoonotic potential and public health impact [18].

*C. argus* is a highly prized freshwater fish in China due to its taste, high nutritional content, and medicinal properties [19]. Nonetheless, bacterial infections pose a significant threat to *C. argus* farming, including pathogens such as *Aeromonas veronii* [20], *Nocardia seriolae* [21], and *Edwardsiella tarda* [22]. *P. shigelloides* can be found in ponds, rivers, streams, and aquatic animals, particularly fish, which serve as its primary host. Phylogenetic analysis utilizing the *16S rRNA* gene confirmed the identity of strain CA-HZ1 as *P. shigelloides*, demonstrating clear clustering within a clade consisting solely of established *P. shigelloides* strains (Figure 2A). This finding is corroborated by the independent *gyrB*-based phylogeny (Figure 2B), solidifying the species assignment. While this places CA-HZ1 firmly within the *P. shigelloides* species complex, the current analysis, reliant on single marker genes, provides resolution sufficient for species identification but does not elucidate the finer-scale evolutionary position of CA-HZ1 within *P. shigelloides* subclades. However, defining CA-HZ1’s specific position within *P. shigelloides* lineages and tracing its exact transmission pathways require substantial further investigation.

Antimicrobial resistance in bacteria represents one of the most critical threats to global public health, jeopardizing effective clinical management of infectious diseases. Bacterial genome sequencing technology facilitates in-depth analysis of resistance mechanisms within aquatic ecosystems. *P. shigelloides* strain CA-HZ1 demonstrates significant ecological and regulatory concern through its resistance to eight antibiotics, despite its prohibition in aquaculture (Table 3). Crucially, persistent resistance determinants (225 identified ARGs) harbored on a mobile genetic element (311,332 bp plasmid) foster reservoirs for horizontal gene transfer to environmentally pervasive pathogens (Figure 3). This compromises approved aquaculture antibiotics via co-selection mechanisms, wherein resistance genes linked to banned antimicrobials commonly co-localize with genes conferring resistance to licensed aquaculture agents on shared plasmids. Moreover, detection of such resistance signatures in foodborne pathogens like CA-HZ1 may invoke international trade barriers, as multidrug-resistant strains could persist during processing, thereby posing contamination risks [23]. Therefore, addressing antimicrobial resistance is not only a matter of aquaculture sustainability but also a central objective for closely integrating the health and welfare of animals, humans, and ecosystems. In Gram-negative bacteria, the acquisition of antibiotic resistance—particularly through point mutations or horizontal gene transfer—is frequently associated with a metabolic burden, as cellular resources are reallocated to resistance-related processes such as the production of resistance enzymes, activation of efflux pumps, or remodeling of membrane components. This additional cost is theoretically expected to compromise growth efficiency and modulate virulence expression. Nevertheless, studies have demonstrated that certain resistance mechanisms in *Pseudomonas aeruginosa* do not necessarily impose a detectable fitness disadvantage. For example, resistant mutants overexpressing the MexEF-OprN efflux system achieved metabolic compensation by activating the anaerobic nitrate respiratory chain, thereby maintaining fitness comparable to the wild type [24]. Similarly, NalD mutations in naturally resistant isolates, which drive constitutive expression of the MexAB-OprM efflux pump, conferred drug resistance without detectable fitness loss after antibiotic withdrawal [25]. These findings indicate that resistance does not invariably incur a biological cost. In parallel, antibiotic-induced metabolic reprogramming—including alterations in glycolysis, tricarboxylic acid (TCA) cycle activity, fermentative pathways, and redox homeostasis—has been closely linked to both resistance and tolerance. A recent review in the International Journal of Molecular Sciences emphasized that multiple classes of antibiotics can remodel central metabolic pathways to promote bacterial persistence and tolerance, thereby setting the stage for the eventual acquisition of stable resistance [26]. Such metabolic plasticity may transiently suppress virulence factor expression, leading to an elevated LD_50_; however, once resistance determinants are stably integrated into the regulatory network, pathogenicity can be restored or, in some cases, further enhanced. Tests for experimental infection have shown that *P. shigelloides* exhibits strong pathogenicity towards *C. argus*. Hu et al. reported a *P. shigelloides* isolate from diseased *C. idella* exhibiting an LD_50_ of 6.4 × 10^4^ CFU/g [7]. This value is 3.5-fold higher than CA-HZ1’s LD_50_ (1.83 × 10^4^ CFU/g) in *C. argus*, indicating superior virulence. Comparative data indicate that *C. carpio* infected with *P. shigelloides* strain XX239 reached a mortality rate of 100% at a bacterial concentration of 1.0 × 10^7^ CFU/mL [27]. *C. carpio* infected with *P. shigelloides* Cc2021 showed 80% mortality at 1.0 × 10^8^ CFU/mL [8], whereas CA-HZ1 caused 96.7% mortality in *C. argus* at a comparable dose (2.16 × 10^8^ CFU/mL). These data confirm the exceptionally high lethality of CA-HZ1 in its novel piscine host. The variation in LD_50_ values among different studies suggests that there are variations in the pathogenicity of different isolates of *P. shigelloides*. Additionally, the present work has reported histological alterations of *C. argus* infected by *P. shigelloides*, with significant alterations observed in the liver, kidney, spleen, and intestine (Figure 5) in comparison with healthy fish. These findings indicate that the CA-HZ1 strain is a virulent isolate, which has greatly threatened the aquaculture industry.

Changes in immune-related gene expression post-infection are critical indicators of the immune response in *C. argus*. *IL-6*, *IL-1β*, *IL-21*, *STAT1*, and *HSP70* were selected as representative immune genes in *C. argus* because they comprehensively cover key immune pathways, including inflammation regulation, lymphocyte activation, chemokine induction, stress protection, and interferon defense, providing a holistic view of the host response to bacterial infection (Figure 6). *IL-6* orchestrates inflammatory responses through dual-phase regulation, while *IL-21* mediates lymphocyte activation via the conserved JAK-STAT pathway. Specifically, Li’s study confirmed IL-21-dependent protection against pathogens in snakehead [28]. Notably, *IL-1β* triggers chemokine cascades in fish macrophages [29], *HSP70* is important for synergistic immunity and stress responses [30], and *STAT1* drives interferon-mediated defenses in teleosts [31]. Our study observed significant upregulation of *IL-6*, *IL-1β*, *IL-21*, *STAT1*, and *HSP70* in *P. shigelloides*-infected *C. argus*, demonstrating pathogen-induced activation of innate immune and inflammatory responses.

The liver functions as the primary organ for detoxification processes, biosynthesis, and cellular metabolism. To investigate the regulatory mechanisms through which *P. shigelloides* influences hepatic inflammation in the northern snakehead, this study utilized RNA sequencing (RNA-seq) to conduct a comprehensive analysis of the liver transcriptome. The DEGs identified were predominantly associated with biological processes related to the immune system, signal transduction, and cellular processes. In a study by Wu et al., a GO analysis was conducted on the intestines of *C. argus* infected with *Aeromonas hydrophila*, revealing that cellular processes were among the most prevalent categories within biological processes [11]. After injecting *A. hydrophila* into *C. argus*, Wang et al. observed a notable enrichment of the signal transduction-related pathways in *C. argus* following *A. hydrophila* injection [32]. Consistent with these findings, our study also revealed a comparable enrichment of signal transduction-related pathways in the livers of the two experimental fish groups.

Recent studies indicate that the *MAPK* signaling pathway plays pivotal roles in teleost physiological adaptations, including thermal acclimation, osmoregulation, hypoxia stress response, and immune defense. Upon *Aeromonas hydrophila* infection in the head kidney of *Larimichthys crocea*, Zhang et al. observed significant upregulation of *MAPKK* genes, demonstrating this pathway’s active regulation of immune responses and environmental adaptation. Similarly, infection with *Pseudomonas plecoglossicida* induced elevated expression of MAPKK4b and MAPKK7 in the spleen within 24 h, corroborating MAPK’s regulatory mechanism in antibacterial immunity [33]. Transcriptomic analyses revealed that *Nocardia seriolae* infection triggers differential MAPK expression across *C. argus* tissues, including gills, accessory respiratory organs, brain, and spleen, highlighting its central role in orchestrating stress responses and innate immunity [34]. Concurrently, the major histocompatibility complex (MHC), a core component of piscine adaptive immunity, enables highly specific immune responses against pathogens. *MHC* gene polymorphism and genomic arrangement critically determine antigen recognition efficacy, making the investigation of MHC interactions with *P. shigelloides*-induced pathologies essential for developing targeted disease prevention strategies [35]. Following *P. shigelloides* infection in *C. argus*, the antigen processing and presentation pathway was significantly enriched with differentially expressed genes. This immune response is predominantly mediated by pattern recognition receptors (PRRs), which detect pathogen-associated molecular patterns (PAMPs) on invading microbes. *HSP70* functions as an endogenous adjuvant, facilitating peptide loading onto MHC class I molecules within antigen-presenting cells (APCs) [36]. Extracellularly, *HSP70*–peptide complexes bind scavenger receptors on neighboring APCs. Therefore, the increased transcription level of *HSP70* in infected *C. argus* enhances cross-presentation and downstream immune activation. In order to activate the immune response of *C. argus*, the antigen must be processed and presented to lymphocytes in the context of MHC molecules expressed on the surface of antigen-presenting cells. At the same time, it is speculated that *HSP70* released from *C. argus* binds to surface receptors such as *TLR2* and *TLR4*, and the damage-related molecular pattern stimulates a variety of immune cells to protect the body from *P. shigelloides*, which is similar to the immune response mechanism of other fish under external stress [37]. Endoplasmic reticulum-resident CALR coordinates MHC class I antigen presentation by chaperoning peptide loading, ensuring proper folding of *MHC-I* complexes, and quality-controlling their surface expression [38]. The upregulation of CALR reflects the increased demand for antigen presentation by host cells and the rapid immune response to pathogen challenge. *HSP90* maintains the correct folding and stability of proteins mainly through ATP-dependent mechanisms and assists in the functions of various signal transduction molecules. In the process of *C. argus* infection of *P. shigelloides*, *HSP90* ensures the processing and presentation efficiency of antigenic peptides by regulating immune-related signaling pathways and regulates the stability of signal molecules through the chaperone network, thereby enhancing the anti-infection ability of *C. argus* [39]. Complementarily, upregulated lysosomal cathepsins (CTSB/L/S) mediate proteolytic cleavage of internalized antigens, generating epitopes for *MHCII* presentation to CD4+ T cells [40]. In summary, these molecular mechanisms highlight coordinated endoplasmic reticulum–lysosomal collaboration to regulate antigen processing, enhancing immunogenicity and ensuring precise defense against *P. shigelloides*. These findings underscore the robust immune response elicited in *C. argus*.

## 5. Conclusions

This study characterizes a hypervirulent *P. shigelloides* strain from an emerging vertebrate host, revealing a complete genome assembly with essential virulence determinants encoded within its 3.49 Mb chromosome and 311 kb plasmid. The findings significantly extend the known host tropism of this pathogen while demonstrating its capacity to dysregulate core immune pathways. Liver transcriptomics further elucidated *P. shigelloides* manipulation of host cellular homeostasis through conserved molecular mechanisms. These results elucidate the molecular basis for its hypervirulence and highlight its potential threat as an emerging pathogen across multiple species. The genomic and transcriptomic resources provided here establish a critical foundation for future functional studies targeting specific virulence mechanisms and for developing targeted surveillance and intervention strategies.

## Figures and Tables

**Table 1:** Primers utilized in qRT-PCR.

Gene Name	Forward Primer (5′-3′)	Backward Primer (5′-3′)	Size (bp)	Accession No.
*IL-6*	CAGGTGATGAGGAGGTGGAG	TGAAGTTGGAGGCAGGACAT	186	XM_067503143.1
*IL-1β*	GACACGATGCGATTCCTATTCT	CACTGGGCAGTCTTCTCGGA	143	XM_067519725.1
*IL-21*	ATATTGAGGACTGCTGCT	TGACTTGTAAGGCTTCTGT	115	XM_067519487.1
*STAT1*	AAGCACCTCCTCTCCAACTC	ACACAGCCTTGACTTTGAGC	163	XM_067501576.1
*HSP70*	TGTCATGGATGCAGCTCAGA	AGACTGACACCTGGTAACCG	173	XM_067492141.1
*β-actin*	GTCTTCCCCTCCATCGTCG	TGGTCACAATACCGTGCTCG	145	XM_067476706.1

**Table 2:** Physiological and biochemical features of isolates.

Characteristic	*P. shigelloides* CA-HZ1	Characteristic	*P. shigelloides* CA-HZ1
Arabinitol	−	Mannitol	−
Glucose	+	Sucrose	−
Arabinose	−	L-RhaMnose	−
Hydrogen sulfide	−	Esculin	−
Citrate	−	Malonate	−
V-P test	−	Maltose	+
D-Xylose	−	Inositol	+
Nitrate reduction	+	Oxidase	+
Indole	+	Dulcitol	−
Sorbitol	−	Urea	−

**Table 3:** Antibiotic susceptibilities of *P. shigelloides* CA-HZ1 against 35 antimicrobial agents.

Antibiotic	Concentration (μg/Piece)	Test Diameter of the Inhibition Zone (mm)	Sensitivity
Amikacin	30	15	I
Gentamicin	120	19	S
Tobramycin	10	20	S
Kanamycin	30	11	R
Streptomycin	10	13	I
Erythromycin	15	7	R
Medemycin	30	6	R
Norfloxacin	10	27	S
Levofloxacin	5	31	S
Ofloxacin	5	32	S
Ciprofloxacin	5	32	S
Polymyxin B	300	15	S
Clindamycin	2	6	R
Clarithromycin	15	11	R
Nitrofurantoin	300	26	S
Tetracycline	30	27	S
Aztreonam	30	20	S
Minocycline	30	27	S
Penicillin	10 u	13	R
Oxacillin	1	0	R
Ampicillin	10 u	25	S
Spectinomycin	100	21	S
Piperacillin	100	0	R
Cefoxitin	30	25	S
Cefazolin	30	25	S
Ceftofur	30	20	S
Cefotaxime	30	27	S
Cefepime	30	25	S
Cefuroxim	30	29	S
Ceftazidime	30	24	S
Ceftriaxone	30	19	I
Cefoperazone	75	25	S
Vancomycin	30	21	S
Pediatric compound sulfamethoxazole tablets	23.75/1.25	23	S
Chloramphenicol	30	35	S

## Data Availability

The original contributions presented in the study are included in the article; further inquiries can be directed to the corresponding author.

## Supplementary Materials

The following supporting information can be downloaded at: https://www.mdpi.com/article/10.3390/microorganisms13092168/s1, Table S1: Classification of virulence factors; Table S2: Summary of predicted antibiotic resistance genes by category; Table S3: Detailed list of predicted antibiotic resistance genes.
